# Low serum vitamin D levels in type 2 diabetes patients are associated with decreased mycobacterial activity

**DOI:** 10.1186/s12879-017-2705-1

**Published:** 2017-09-07

**Authors:** María Teresa Herrera, Yolanda Gonzalez, Fernando Hernández-Sánchez, Guadalupe Fabián-San Miguel, Martha Torres

**Affiliations:** 1 Departamento de Investigación en Microbiología, Instituto Nacional de Enfermedades Respiratorias “Ismael Cosío Villegas”, Calzada de Tlalpan 4502, 14080 Ciudad de México, Mexico; 2 Clínica del Síndrome Metabólico, Instituto Nacional de Enfermedades Respiratorias “Ismael Cosío Villegas”, Calzada de Tlalpan 4502, 14080 Ciudad de México, Mexico

**Keywords:** Type 2 diabetes mellitus, Vitamin D, Human monocytes, Tuberculosis, *Mycobacterium tuberculosis*

## Abstract

**Background:**

Concurrent diabetes mellitus and tuberculosis represent a significant health problem worldwide. Patients with diabetes mellitus have a high risk of tuberculosis, which may be mediated by an abnormal innate immune response due to hyperglycaemia or low vitamin D levels.

**Methods:**

In the present study, we evaluated inactive vitamin D serum levels and the monocyte response to infection with *M. tuberculosis*, including phagocytosis of *M. tuberculosis*, antimycobacterial activity, LL-37, human β defensin-2 and IL-10 gene expression and nitric oxide production, between type 2 diabetes mellitus patients (*n* = 51) and healthy volunteers (*n* = 38).

**Results:**

Twenty-seven type 2 diabetes mellitus patients had inadequate inactive vitamin D levels (<50 nM). The percentages of *M. tuberculosis* phagocytosis between monocytes were similar across groups according to microscopy. Intracellular mycobacterial growth was similar in infected monocytes from both groups. However, *M. tuberculosis* growth was significantly higher in monocytes obtained from type 2 diabetes mellitus patients and lower vitamin D levels after 1-h (D0) and 72-h (D3) post-infection (*p* ≤ 0.05). LL-37, human β defensin-2 and IL-10 mRNA expression were similar between monocytes across groups; vitamin D serum levels and LL-37, human β defensin-2 and IL-10 expression were not correlated. Nitric oxide production was significantly higher in healthy volunteers than in type 2 diabetes mellitus patients with low vitamin D serum levels at D3 post-infection (*p* ≤ 0.05).

**Conclusions:**

Our results show that monocytes from type 2 diabetes mellitus patients and low vitamin D serum levels show an impaired ability to control the intracellular growth of *M. tuberculosis*, which is not associated with significant decrease of LL-37 or human β defensin-2 expression. Vitamin D could be the link between diabetes and tuberculosis susceptibility.

**Electronic supplementary material:**

The online version of this article (10.1186/s12879-017-2705-1) contains supplementary material, which is available to authorized users.

## Background

Vitamin D insufficiency is a risk factor for osteomalacia in adults and rickets in children and is associated with a variety of conditions, such as various cancers, type 1 and 2 diabetes, hypertension, multiple sclerosis, metabolic syndrome and infectious diseases, including tuberculosis [1–4].

The human body can synthesize vitamin D from the precursor molecule 7-dehydrocholesterol, which is generated by the skin upon sufficient exposure to solar ultraviolet radiation. Vitamin D is also obtained from natural food, particularly from fish. Since the final effector molecule of vitamin D is produced in humans, vitamin D is also considered a pro-hormone [5]. Vitamin D status is determined by measuring circulating 25-hydroxyvitamin D (25OHD_3_), which is the predominant inactive form in plasma or serum and is the first hydroxylation product of vitamin D that is synthesized in the liver.

Currently, there is a debate regarding the definition of vitamin D insufficiency. Although the majority of Working Group members nominated 75 nM as the appropriate target concentration for older individuals in the International Osteoporosis Foundation position statement [6]. However, other groups have opted for a lower target of 50–75 nM. The North American Institute of Medicine has classified serum 25OHD_3_ levels of 50 to 125 nM as sufficient, levels between 30 and 49.99 nM as inadequate, levels below 30 nM as deficient and levels above 125 nM as potentially harmful [6].

The active metabolite of vitamin D, 1,25-dihydroxyvitamin D3 (1,25(OH)_2_D_3_), influences pancreatic β-cells and insulin secretion [7]. Although a systemic review and meta-analysis that included clinical trials on the effect of vitamin D on glycaemic control in persons with diabetes mellitus (DM) have not shown an effect, previous reports have shown a clear association between low levels of vitamin D and DM.

In persons with established DM and in the general population, low levels of 25OHD_3_ are associated with higher fasting glucose, insulin resistance and metabolic syndrome [8].

Vitamin D plays a prominent role in innate immunity, and vitamin D receptors (VDR) are found on monocytes; these cells differentiate into macrophages under the influence of 1,25(OH)_2_D_3_ [9]. The vitamin D also is active in many other cells, such as keratinocytes, epithelial cells and natural killer cells [10, 11]. Vitamin D deficiencies are known to affect host immunity [12]. Vitamin D exerts regulatory activity to modulate memory and effector CD8+, CD4+, regulatory T cells and regulation of cytokines [3, 13]. In macrophages, this vitamin modulate phagocytosis and autophagy between others mechanism [14]. The antimicrobial response induced by Vitamin D involves recognition of bactericidal lipoproteins by Toll-like receptors (TLRs), induction of the 25-hydroxyvitamin D3–1α-hydroxylase (CYP27B1), which converts the vitamin D prohormone (25OHD_3_) into the active 1,25(OH)_2_D_3_ form and the activation of the VDR that directly modulate gene expression that favours antimicrobial peptides production [15].

Vitamin D restricts intracellular *Mycobacterium tuberculosis* replication via the induction of antimicrobial peptides such as cathelicidin (LL-37) and human β defensin-2 (HBD2) [15, 16]. Previous studies have reported that 1,25(HO)_2_D_3_ induces differentiation of the human monocytic THP-1 cell line and enhances the chemotactic and phagocytic activity of macrophages [17, 18]; 1,25(HO)_2_D_3_ also plays a role in acquired immunity directly by acting on T cells and indirectly by regulating dendritic cells; it also restricts Th1/Th17 cell differentiation and favours Th2 response [10].

In addition, previous research has shown that DM triples the risk of tuberculosis (TB) [19]. Several studies have shown an association between low levels of vitamin D and both TB and DM [7, 20, 21]

The high prevalence of DM in Mexico results in a considerable proportion of TB cases attributable to this disease [19]. The susceptibility to *M. tuberculosis* infection observed in persons with DM suggests an impaired immune response, and vitamin D could be the link between DM and TB susceptibility [22].

Therefore, in this work, we evaluate the correlation between serum levels of vitamin D in type 2 diabetes mellitus (T2D) patients and antimycobacterial activity.

## Methods

### Participants

Fifty-one T2D patients (17 man/34 woman) and 38 people healthy volunteers (12 man/26 woman) were enrolled in this study from 2008 to 2015. The T2D patients were invited to participate in the study at the Metabolic Syndrome Clinic of the National Institute of Respiratory Diseases in Mexico City. They were diagnosed according to the criteria of the American Diabetes Association (Diagnosis and Classification of DM) [23], and active TB was ruled out by clinical and chest X-ray findings. Additionally, healthy volunteers were invited to participate in this study. All participants provided written informed consent, and routine laboratory tests on blood, as well as chest X-rays and PPD tests, were conducted. The Ethics Committee of the National Institute of Respiratory Diseases approved this study.

### Determination of vitamin D

Blood samples were drawn from all participants, and the serum samples were separated and stored at −80 °C until determination of the inactive form of vitamin D (25OHD_3_). Serum 25OHD_3_ levels were determined using a commercial serum vitamin D kit (Liaison, Diasorin MN, USA) according to the manufacturer’s instructions and reported as nM.

### Monocyte isolation

Sixty millilitres of heparinized human peripheral venous blood was obtained from the participants, and peripheral blood mononuclear cells were obtained from whole blood by centrifugation using Lymphoprep® (Nycomed Pharma, Oslo, Norway) [24].

Monocytes were enriched by positive selection using MACS® magnetic beads coupled to anti-human CD14 antibodies (Miltenyi Biotech, Auburn, CA), according to the manufacturer’s recommendations. The purity of monocytes was assessed by conventional flow cytometry using anti-human CD14 antibodies. The cell preparations were routinely >95% pure, and viability was evaluated using the trypan blue exclusion assay, which revealed 99% viability.

### Media and cell culture

Monocytes were cultured in RPMI 1640 (Cambrex, Walkersville, MD) supplemented with 50 μg/ml gentamicin sulfate, 2 mM L-glutamine and 10% heat-inactivated pooled human serum (Gemini Bioproducts, Sacramento, CA) at 37 °C under a 5% CO_2_ atmosphere.

### Preparation of *M. tuberculosis* H37Ra

The *M. tuberculosis* strain H37Ra (ATCC # 25177, Manassas, VA) was prepared in Middlebrook 7H9 broth medium (Difco Laboratories, Detroit, MI) supplemented with 10% albumin-dextrose-catalase (ADC, Difco Laboratories) and 0.2% glycerol. The mycobacteria were incubated for 21 days at 37°C with 5% CO_2_ on an orbital shaking incubator, and the *M. tuberculosis* suspension was harvested, centrifuged (1972 g, 15 min, 25 °C), resuspended in fresh 7H9 medium and aliquoted into one-millilitre samples that were stored at −70 °C until use. The mycobacteria stock concentration was determined by colony forming units (CFU) counts from ten-fold serial dilutions in 7H9 medium after 21 days of incubation on 7H10 agar. For infection assays, *M. tuberculosis* stock was thawed and centrifuged (5220 g, 8 min, 25 °C), and the supernatant was replaced by complete medium (supplemented medium with 10% non-heat-inactivated human pool serum). Then, mycobacteria were declumped by mixing and repeatedly forcing the sample out of a 27-G syringe needle, followed by a 1-min sonication (Untrastonic 28X, Ney Dental International, Yacaipa, CA) and centrifugation (82 g, 2 min, 25 °C). The supernatant containing disaggregated mycobacteria was recovered and used in infection assays.

### Phagocytosis assay by microscopy

A total of 1 × 10^5^ monocytes/well were placed in a chamber slide (Nunc-Thermo Fisher Scientific, Rochester, NY) and were allowed to adhere for 1 h at 37 °C in 5% CO_2_. The cells were subsequently incubated with either culture medium (RPMI 1640, 10% pool human serum) or infected with *M. tuberculosis* at multiplicity of infection (MOI) 10 (10 mycobacteria: 1 monocyte) and incubated for 1 h. Three washes with supplemented RPMI were performed to remove the extracellular mycobacteria, and the cells were fixed for 10 min with methanol. Then, the slide was stained with a TB stain kit-K (Becton-Dickinson, San Jose, CA) and analysed under a microscope (Carl-Zeiss, Gottingen,Germany) at 100X with immersion oil. We counted 300–400 monocytes, and the number of monocytes with intracellular mycobacteria was expressed as the phagocytosis percentage.

### *Mycobacterium tuberculosis* infection and intracellular mycobacterial growth

A total of 2 × 10^5^ monocytes/well were infected with *M. tuberculosis* at MOI of 10 in a 96-well culture plate (Corning Costar Co., Corning, NY). Subsequently, the infected monocytes for 1 h (D0), 24 h (D1) and 72 h (D3) and mycobacterial intracellular growth was evaluated by serial dilution and CFU counts.

### Quantifying CFU

The intracellular growth of *M. tuberculosis* was assessed by CFU. Briefly, the infected monocytes at D0, D1 and D3 were lysed as previously reported [25]. Ten-fold serial dilutions of the triplicates were performed, and the dilutions were seeded on 7H10 agar plates in triplicate. After 21 days at 37 °C under a 5% C0_2_ atmosphere, the CFU were counted.

### Gene expression by real-time quantitative PCR

After a 1-h (D0) or 72-h (D3) incubation, the uninfected (UN) and infected monocytes (*M.tb*) were lysed, and total RNA was extracted by column purification using a RNeasy Mini Kit (Qiagen Co., Strasse, Germany) following the manufacturer’s instructions. RNA was eluted in DEPC-water and used for cDNA synthesis with a Superscript First-Strand-cDNA Synthesis System (Invitrogen, Carlsbad, CA). Briefly, RNA with random hexamers and dNTPs was denatured (65 °C, 5 min), and a reverse transcription mix (25 mM MgCl_2_, 2 mM DTT, RNAse Out inhibitor, PCR buffer 10X) was added. The synthesis was performed with Superscript II enzyme in a thermal cycler (iCycler, BioRad Co.). Quantitative real-time-PCR (qPCR) was performed using Taqman assays for LL-37 (CAMP, Hs00189038, Applied Biosystem, Foster City, CA), human IL-10 (Hs00961622_m1, Applied Biosystem) and ribosomal RNA 18S (rRNA 18S) as a housekeeping control was used for the normalization (Applied Biosystem). Briefly, a universal master mix (Applied Biosystem), TaqMan assay (LL-37-FAM or IL-10-FAM and rRNA18S -VIC), DEPC-water and cDNA were mixed and added to each well of a 96-well plate. The plates were sealed and centrifuged (300 g, 5 min). Amplification was performed in a StepOne Plus Sequence Detection System (Applied Biosystem) starting with 2 min at 50°C, 10 min at 95°C and 40 cycles of 30 s at 95°C and 60 s at 62°C. Data were analysed with 7500 Fast System SDS software version 1.4 (Applied Biosystem), and the results are reported as fold changes respective to UN monocytes. A relative quantification method (∆∆Ct) was used for gene expression analysis.

### Gene expression by PCR

A 102 bp fragment of human β defensin-2 (HBD2) was amplified using the primers 5′-CAT CAG CCA TGA GGG TCT-3′(forward) and 5′-AGG CAG GTA ACA GGA TCG-3′ (reverse) and a fragment of 496 bp from Hypoxanthine Phosphoribosyl Transferase (HPRT) as internal control using the primers 5′-TAT GGA CAG GAC TGA ACG TCT TGC- 3′(forward) and 5′-GAC ACA AAC ATG ATT CAA ATC CCT CA-3 r´(reverse). cDNA were mixed with PCR buffer, MgCl_2_ (3 mM), dNTPs (0.4 mM/each one), primers (0.4 μM and the amplifications were done in a thermocycler by 35 cycles: 30 s of denaturation at 95 °C, 30 s of annealing primers at 55 °C (HBD2) or 60 °C (HPRT) and 1 min of extension at 72 °C. The amplification products were mixed with GelRed and analyzed on 2% agarose gel electrophoresis and the imagen captured in a ChemiDoc MP Imaging System (BioRad, Hercules, CA). The amplification band fluorescence was analyzed with Image Lab software and results were reported as arbitrary units.

### Nitric oxide (NO) determination

After monocytes were infected with *M. tuberculosis*, the supernatants were recovered at 1 h (D0) and 72 h (D3) post-infection, and the NO concentration was determined as previously reported [26]. Briefly, the culture supernatant was added to a 96-well plate and mixed with Griess reagent (0.1% naphtyl-ethilenediamine dihydrochloride and 1% sulfanilamide in 1NHCl) and incubated for ten min. A standard curve with different NaNO_2_ concentrations was included, and the optical density was determined at 550 nm with a plate microreader (Labsystems Multiskan MCC/340; Labsystems, Helsinki, Finland). The NO concentration was reported in μM.

### Statistical analysis

Comparisons between T2D patients and healthy volunteers were made using nonparametric Mann-Whitney Rank Sum test, Wilcoxon signed rank tests or Fisher exact test. For rank correlations, we used Spearman’s rank correlation coefficient on nonparametric data. For PPD, gender and the recruitment of people in the two groups during years and seasons, we used proportion tests. Statistical significance was defined as *p* ≤ 0.05. We used GraphPad Prism Software package, version 5.04 (2008). All data are expressed as the median.

## Results

The clinical characteristics of the study groups are presented in Table 1. The T2D patients was significantly older and had higher levels of fasting glucose, glycated haemoglobin A (HbA1c), cholesterol, LDL and triglycerides than the group of healthy volunteers (*p* ≤ 0.05). Most T2D patients were taking metformin and glibenclamide, but none were prescribed angiotensin receptor blockers, insulin, or statins. Healthy volunteers did not exhibit any infections or inflammatory diseases at the time of blood donation for the experiments and did not take any medications during the study period. Complementary clinical characteristic and data base are presented in Additional file 1: Table S1 and S2.

**Table 1:** The clinical description of the study groups

	T2D	Healthy	*p ≤ 0.05
n	51	38	
Gender (Men/Women)	17/34	12/26	ns
Age (years)	49.4 ± 7.7	44.8 ± 8.0	*
Weight (kg)	69.4 ± 12.3	66.6 ± 10.3	ns
Height (m)	1.58 ± 0.08	1.59 ± 0.08	ns
BMI (Kg/m^2^)	27.4 ± 4.5	26.0 ± 2.7	ns
Fasting glucose (mg/dL)	206.6 ± 82.9	95.3 ± 7.9	*
HbA1c (%)	9.4 ± 2.5	5.5 ± 0.2	*
Creatinine (mg/dL)	0.73 ± 0.15	0.79 ± 0.15	ns
Cholesterol (mg/dL)	208.9 ± 40.0	179.4 ± 23.4	*
HDL (mg/dL)	42.3 ± 9.1	43.6 ± 10.7	ns
LDL (mg/dL)	128.8 ± 34.7	111.9 ± 18.4	*
Triglycerides (mg/dL)	271.3 ± 213.2	137.6 ± 46.5	*
Status PPD (+/−)	56.8% (29/22)	57.8% (22/16)	ns

*T2D* Type 2 diabetes mellitus, *BMI* Body mass index, *HbA1c* Glycated haemoglobin, *HDL* High density lipid, *LDL* Low density lipid, *ns* no significant

Values represent Mean **±** SD, T test, T2D vs. Healthy. Two-sample proportion test for PPD and Gender

### Vitamin D levels in T2D patients

**Fig. 1:**
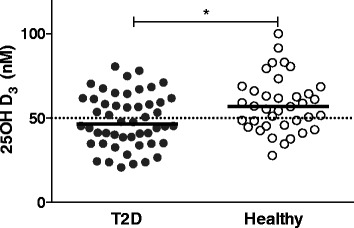
Serum levels of vitamin D in T2D patients and healthy volunteers. The inactive form of vitamin D (25OHD_3_) was measured in serum samples from the studied groups by a chemiluminescence assay, and the concentration is reported in nM. The dot graph shows the serum levels of 25OHD_3_ in T2D patients, *n* = 50, (*closed circles)* and Healthy volunteers, *n* = 37, (*open circles*). *p ≤ 0.05, T2D vs. Healthy (Mann-Whitney U test). The median is the horizontal line

Since the levels of vitamin D could have seasonal variations, we searched for seasonal differences among samples of T2D patients and healthy volunteers obtained at various times, and we did not find any differences (p>0.05). The serum levels of vitamin D in the T2D patients and healthy volunteers groups were significantly different. Our results show that 54% of T2D patients had an inadequate level of vitamin D (between 30 and 49.99 nM) (Fig. 1).

### *M. tuberculosis* phagocytosis in monocytes from T2D patients

**Fig. 2:**
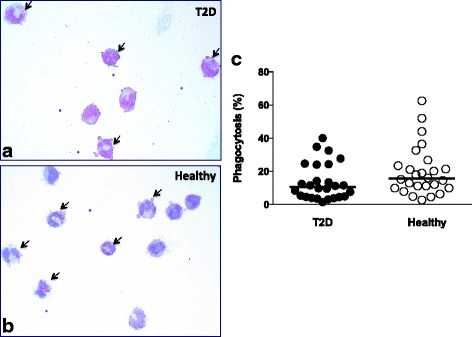
Phagocytosis of *M. tuberculosis* by monocytes from T2D patients and healthy volunteers. A total of 1 × 10^5^ monocytes/well were placed in a chamber slide and adhered during a 1-h incubation at 37 °C, 5% CO_2_. Monocytes were infected with *M. tuberculosis* at MOI 10.. Phagocytosis was carried out during a 1-h incubation, and then extracellular *M. tuberculosis* was eliminated by washing and fixing the cells. Monocytes were stained and analysed with microscopy at 100X with immersion oil. In total, 300–400 monocytes were counted, and monocytes with intracellular *M. tuberculosis* were reported as the phagocytosis percentage. Representative images show the phagocytosis of *M. tuberculosis* in monocytes from **a** T2D patients and **b** Healthy volunteers; (the *arrows* indicate the monocytes with intracellular *M. tuberculosis*). **c** The *dot* graph shows the phagocytosis by monocytes from T2D patients, *n* = 26, (*closed circles)* and Healthy volunteers, *n* = 26, (*open circles)*. *p* > 0.05, T2D vs. Healthy (Mann-Whitney U test). The median is the horizontal line

Vitamin D plays a role in innate immunity and has been described to affect mycobacterial phagocytosis [27, 28]. Therefore, we analysed the phagocytosis of *M. tuberculosis* by monocytes from T2D patients and healthy volunteers. Our results showed that the percentages of monocytes that phagocytosed *M. tuberculosis* were similar (*p*>0.05) in T2D patients (Fig. 2a) and healthy volunteers (Fig. 2b), and phagocytosis was not associated with levels of vitamin D (Fig. 2c).

### Intracellular growth of *M. tuberculosis* was higher in monocytes from T2D patients and low vitamin D levels

**Fig. 3:**
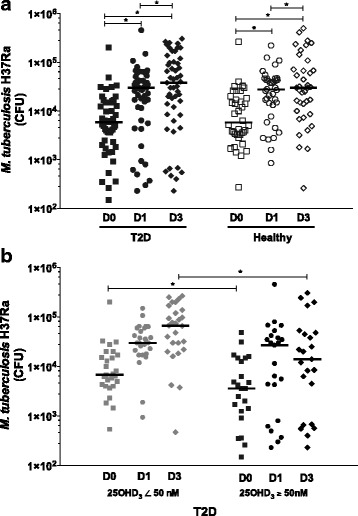
Intracellular growth control of *M. tuberculosis* by monocytes from T2D patients and healthy volunteers. A total of 1 × 10^5^ monocytes/well were placed in a 96-well plate and incubated for 1 h at 37 °C, 5% CO_2_. The monocytes were infected with *M. tuberculosis* at MOI 10 for 1 h, and extracellular *M. tuberculosis* was eliminated by washing. After 1 h (D0), 24 h (D1) and 72 h (D3) of incubation, the supernatants were recovered, the infected monocytes were lysed and *M. tuberculosis* was counted with a CFU assay. **a** The dot graph depicts the *M. tuberculosis* intracellular growth in monocytes T2D patients, *n* = 51, (*closed symbols)*, and Healthy volunteers, *n* = 38, (*open symbols)*. *p ≤ 0.05, T2D and Healthy, D0 vs. D1and D3; and D1 vs. D3 (Wilcoxon test). **b** The dot graph depicts the intracellular growth of *M. tuberculosis* according to the 25OHD_3_ serum levels T2D patients <50 nM, *n* = 27, (*grey symbols)* and ≥50 nM, *n* = 23, (*closed symbols)*. *p ≤ 0.05, 25OHD_3_ < 50 nM vs. ≥50 nM at D0 and D3,(Mann-Whitney U test). In all graphics the median is represented by horizontal line

Then, we explored the intracellular mycobacterial growth in monocytes obtained from T2D patients and healthy volunteers. No significant differences in mycobacterial growth were observed between our groups (*p*>0.05) (Fig. 3a). However, *M. tuberculosis* growth was significantly higher in monocytes obtained from T2D patients and inadequate levels of vitamin D (<50 nM) after 1 h (D0) and 72 h (D3) post-infection (*p* ≤ 0.05) (Fig. 3b), with a significant negative correlation of *r* = − 0.39 and −0.44, respectively.

### LL-37, HBD2 and IL-10 expression and NO production are not associated with serum vitamin D levels

**Fig. 4:**
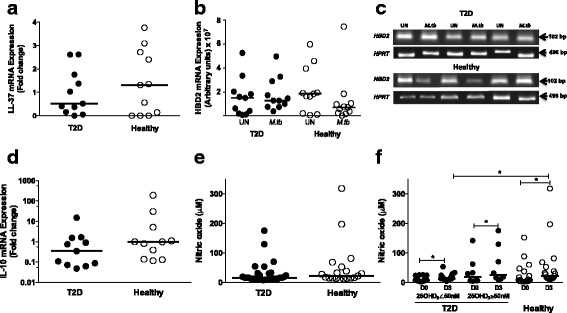
LL-37, HBD2 and IL-10 gene expression and NO production by monocytes infected from T2D patients and healthy volunteers. A total of 0.5 × 10^5^ monocytes/well were placed in a culture plate and infected with *M. tuberculosis* at MOI 10 for 1 h, extracellular *M. tuberculosis* was eliminated by washing, and the monocytes were incubated for 1 h (D0), 24 h (D1) and 72 h (D3). The supernatants were harvested, and the cells lysed for RNA extraction and cDNA synthesis. LL-37 and IL-10 gene expression was assessed using qPCR and HBD2 was analysed by PCR. In the supernatants, the nitrite (−NO_2_) concentration was determined by Griess reagent and reported as μM. **a** The dot graph shows the LL-37 gene expression in T2D patients, *n* = 11(*closed circles)* and Healthy volunteers, *n* = 11(*open circles)* at D3. *p* > 0.5, T2D vs. Healthy (Mann-Whitney U test). **b** The dot graph shows the HBD2 gene expression in T2D patients, *n* = 11 and Healthy volunteers, *n* = 11 in uninfected (UN) and infected (*M.tb*) monocytes at D3. *p* > 0.05, T2D vs. Healthy (Mann-Whitney U test). **c** The image shows representatives amplifications of HBD-2 and HPRT fragments used as housekeeping gene in UN and *M.tb* monocytes from T2D (*n* = 3) and Healthy volunteers (*n* = 3) at D3. **d** The dot graph shows the IL-10 gene expression in T2D patients, *n* = 11 and Healthy volunteers, *n* = 11 at D3. *p* > 0.05, T2D vs. Healthy (Mann-Whitney U test). **e** The dot graph shows the NO production by monocytes from T2D patients, *n* = 30 and Healthy volunteers, *n* = 19 at D3. *p* > 0.05, T2D vs. Healthy (Mann-Whitney U test). **f** NO production by monocytes from T2D patients with levels of 25OHD_3_ < 50 nM, *n* = 22, >50 nM, *n* = 8 and Healthy volunteers, *n* = 19 at D0 and D3. *p ≤ 0.05, T2D, 25OHD_3_ < 50 nM and ≥50 nM and Healthy at D0 vs. D3 (Wilcoxon test); *p ≤ 0.05, T2D, 25OHD_3_ < 50 nM vs. Healthy at D3 (Mann-Whitney U test). In all graphics the median is represented by horizontal line

Vitamin D directly and indirectly regulates the expression of the antimicrobial peptides cathelicidin (LL-37) and human β defensin-2 (HBD2), which exert bactericidal activity [15, 16], Because low sera levels of vitamin D were associated with significantly higher intracellular mycobacterial growth, we decided to evaluate the expression of LL-37 and HBD2 in monocytes from T2D patients with lower sera vitamin D levels in comparison with monocytes from healthy volunteers with adequate serum vitamin D levels. We found that LL-37 (Fig. 4a) and HBD2 (Fig. 4b, Fig. 4c) expression was similar between monocytes from T2D patients with low serum levels of vitamin D and those healthy volunteers (p>0.05). We did not find a correlation between serum levels of vitamin D and LL-37 and HBD2 expression.

Then, we measured IL-10 gene expression in monocytes from T2D patients with low serum levels of vitamin D and healthy volunteers. We found not significant difference on IL-10 expression comparing T2D patients to healthy volunteers neither correlation of IL-10 expression with the levels of vitamin D (p>0.05) (Fig. 4d).

Additionally, vitamin D also participates in the regulation of NO production, which exhibits important antibacterial activity [29]. Our results revealed that NO concentrations produced by infected monocytes from T2D patients and healthy volunteers were similar at D3 (*p*>0.05) (Fig. 4e).

However, NO production was significantly increased in the supernatant of infected monocytes at D3 compared with D0 in T2D patients (Vitamin D <50 nM and ≥50 nM) and healthy volunteers post-infection (*p* ≤ 0.05). NO production was significantly higher in healthy volunteers than in T2D patients with low serum vitamin D levels (< 50 nM) (*p* ≤ 0.05) (Fig. 4f).

## Discussion

T2D patients have increased susceptibility to TB, and previous research has shown that low vitamin D levels are associated with both DM and TB. Because vitamin D is a molecule that exert immunoregulatory activities and it has been described that vitamin D play an important role in the immunity against pathogens as *M.tuberculosis*. We wondered whether vitamin D could be involved in antimycobacterial activity in T2D patients. Thus, we explored the levels of vitamin D and its correlation with phagocytosis of *M. tuberculosis* and antimycobacterial activity in monocytes.

Our results showed that 54% of the study participants with T2D had inadequate sera levels of vitamin D, and these results agree with previous reports. Previous studies have shown that T2D patients are more susceptible to TB [19, 30]. Then, we explored the association between low levels of vitamin D and the phagocytosis and intracellular growth of *M. tuberculosis* in monocytes from T2D patients. Our results showed no significant difference in the percentage of monocytes that phagocytosed *M. tuberculosis* from T2D patients compared with those from healthy volunteers, as determined by light microscopy. These results are inconsistent with a recent report describing a reduced percentage of monocytes from T2D patients associated with *M. tuberculosis*; this report also demonstrated that phagocytosis was strongest under higher autologous serum concentrations (20% versus 5%), and the authors concluded that the association involved complement factors [31]. We evaluated phagocytic activity in enriched monocytes using 10% of a pool of commercial non-autologous serum, and we did not study autologous complement components. However, the differences could be explained by variations in the experimental conditions. In the whole blood system, there may be other molecular and cellular mechanisms that participate in phagocytosis independent of the complement factors evaluated by others [31]. Our results also showed that phagocytosis of *M. tuberculosis* in T2D patients did not correlate with serum levels of vitamin D.

Next, we evaluated antimycobacterial activity in T2D patients and healthy volunteers and observed similar intracellular growth of *M. tuberculosis* between the groups. Since the percentage of infected monocytes was comparable between the groups, the magnitude of intracellular growth was similar. These results are also inconsistent with previous reports showing that monocyte bactericidal function was decreased in patients with poorly controlled non-insulin-dependent DM [32]. Although the T2D patients in this study were treated with metformin or glibenclamide, their disease was poorly controlled, as confirmed by the high HbA1c values. In addition, the mycobactericidal activity observed in T2D patients may be associated with metformin therapy. Previous studies have reported that metformin exhibits bactericidal activity and reduces the deleterious inflammation associated with immune pathology [33, 34].

In addition, we analysed the intracellular growth of *M. tuberculosis* in monocytes from T2D patients and associated it with the levels of vitamin D, we found that the mycobactericidal activity in T2D patients was significantly lower in monocytes from patients with inadequate levels of vitamin D. These data indicate that low levels of vitamin D are associated with the decreased antimycobacterial activity observed in T2D patients. Since vitamin D induces the production of LL-37 and HBD2, antimicrobial peptides with antimycobacterial activity [16, 35, 36], we evaluated the expression of LL-37 and HBD2 and its association with vitamin D levels. However, the expression of LL-37 and HBD2 were not significantly decreased in T2D patients with low serum levels of vitamin D and was not correlated with serum vitamin D levels or antimycobactericidal activity. However, the induction of LL-37 was slightly lower in *M. tuberculosis*-infected monocytes from T2D patients with low serum vitamin D levels. Out of its participation in the regulation of antimicrobial peptides, vitamin D also regulates other human genes trough VDR-mediated response elements (Vitamin D Response Elements) and vitamin D inhibits the production of pro-inflammatory cytokines trough MKP-1 pathway [37]. With this rationale, we assessed IL-10 expression and analysed its correlation with vitamin D levels. We found not significant difference on IL-10 expression comparing T2D patients to healthy volunteers neither correlation of IL-10 expression with the levels of vitamin D. These results are consistent with our previous report where IL-10 levels were similar in T2D patients and healthy volunteers [38]. Other authors studied patients with rheumatoid arthritis and also found no correlation of vitamin D levels and the production of IL-10 [39]. Therefore, our results suggest that the decreased capacity to control the intracellular growth of *M. tuberculosis* by monocytes from T2D patients with low serum levels of vitamin D could be associated with another mechanism regulated directly or indirectly by vitamin D other than LL-37, HBD2 or IL-10.

NO is an innate metabolite that exhibits antibacterial activity, and previous reports have indicated that vitamin D affects the expression of mRNAs encoding inducible NO synthase (iNOS), iNOS protein expression and NO production [40, 41]. Therefore, we evaluated NO production and observed that NO production in response to *M. tuberculosis* infection in monocytes from T2D patients with serum low levels of vitamin D was significantly lower than that in monocytes from healthy volunteers at D3. Although lower NO production is in agreement with the decreased antibacterial activity observed in T2D patients with low serum levels of vitamin D, these results are inconsistent with a previous report showing that vitamin D-induced inhibition of NO release [41]. We speculate low serum levels of vitamin D in T2D patients could correlate with higher NO production. Nonetheless, we must also mention that determination of NO in human cells is not a sensitive assay, and these measures are not completely accurate. In addition, other bactericidal mechanisms directly or indirectly influenced by vitamin D remain functional in monocytes from T2D patients and are the responsible for the observed mycobactericidal activity. These mechanisms should be examined in future studies to define the role of vitamin D in the increased susceptibility of T2D patients to TB development.

## Conclusions

The results of our study demonstrate that vitamin D could be the mediator between DM and TB susceptibility. Since monocytes from T2D patients and low serum levels of vitamin D show an impaired ability to control the intracellular growth of *M. tuberculosis* (even though vitamin D levels are not associated with significant expression of LL-37 and HBD2), other mechanisms directly or indirectly regulated by vitamin D could be involved in the inability of monocytes from T2D patients to control *M. tuberculosis* growth.

## Additional file

Additional file 1: Complementary clinical characteristic and data base. **Table S1**. T2D patients. **Table S2**. Healthy volunteers. (XLSX 42 kb)

## Abbreviations

1,25(OH)_2_D_3_: 1,25-dihydroxyvitamin D3
25OHD_3_: 25-hydroxyvitamin D
CFU: Colony forming units
DM: Diabetes mellitus
HbA1c: Glycated haemoglobin A
HBD2: Human β defensin-2
IL-10: Interleukin 10
iNOS: Inducible NO synthase
*M.tuberculosis*: *M*yc*obacterium tuberculosis*
MOI: Multiplicity of infection
NO: Nitric oxide
T2D: Type 2 diabetes mellitus
TB: Tuberculosis
